# AI-Based Cancer Models in Oncology: From Diagnosis to ADC Drug Prediction

**DOI:** 10.3390/cancers17213419

**Published:** 2025-10-24

**Authors:** Navid Sobhani, Fernanda G. Kugeratski, Sergio Venturini, Raheleh Roudi, Tristan Nguyen, Alberto D’Angelo, Daniele Generali

**Affiliations:** 1 Department of Leukemia and Cancer Biology, University of Texas MD Anderson Cancer Center, Houston, TX 77030, USA; 2 Department of Experimental Therapeutics, University of Texas MD Anderson Cancer Center, Houston, TX 77030, USA; fkugeratski@mdanderson.org; 3 Department of Economic and Social Sciences, Catholic University of Sacred Heart—Cremona Campus, 26100 Cremona, Italy; sergio.venturini@unicatt.it; 4 Department of Radiology, Molecular Imaging Program at Stanford, Stanford University, Stanford, CA 94305, USA; roudi@stanford.edu; 5 Department of Cancer Biology, University of Texas MD Anderson Cancer Center, Houston, TX 77030, USA; tnguyen67@mdanderson.org; 6 Department of Medicine, Northern General Hospital, Sheffield S5 7AT, UK; alberto.dangelo@nhs.net; 7 Department of Medicine, Surgery and Health Sciences, University of Trieste, 34100 Trieste, Italy; dgenerali@units.it

**Keywords:** ADC-AI, explainable AI, federated learning, patient selection methods

## Abstract

**Simple Summary:**

With the blossoming of artificial intelligence (AI), many tasks in society have been simplified, from facial recognition tools for data security to medical devices. While the general population considers AI only as an instrument for simple language-related improvements, they remain unaware of its true potential to tackle highly complex tasks- from piloting spaceships to discovery new targeted therapies for incurable diseases. The oncology field has not remained untouched. Although certain AI models have been hindered due to the relatively extrinsic nature of their systems, new AI models have been developed instead. In fact, models respecting a hundred percent the privacy of patients, while being able to explain the “thought process”, could pave the way for future AI infrastructures across institutions to aid clinicians in their time-honored mission of healing patients.

**Abstract:**

**Introduction** Artificial intelligence (AI) has been influencing the way oncology has been practiced. Major issues constituting a bottleneck are the lack of data for training purposes, confidentiality preventing development, or the absence of transparency in clarifying how models operate to generate decisions. **Novel Models** With explainable AI, trust and utilization barriers among clinicians, researchers, and patients can be removed. With the implementation of federated learning, multiple institutions could contribute to crucial dataset’s learning information. Precise diagnosis and prescription of the right drug are essential in preventing unnecessary life losses, and economic burden to the underling system. **Focus** This review focuses on new AI models that could make medical diagnosis more precise, quicker and convenient, as well as help with the discovery of new drugs and better selection of certain cancer therapies, in particular for those drugs whose results have been inconsistent across different groups of patients. We then speculate on the transformative role AI could play in predicting ADC therapy efficacy. This would ultimately bestow the medical field of an impeccable selection tool. Such colossal methodology, coupled with seeming monitoring of treatment and recovery, may be granting remedies that have been so longed, and deemed necessary.

## 1. Introduction

**Figure 1:**
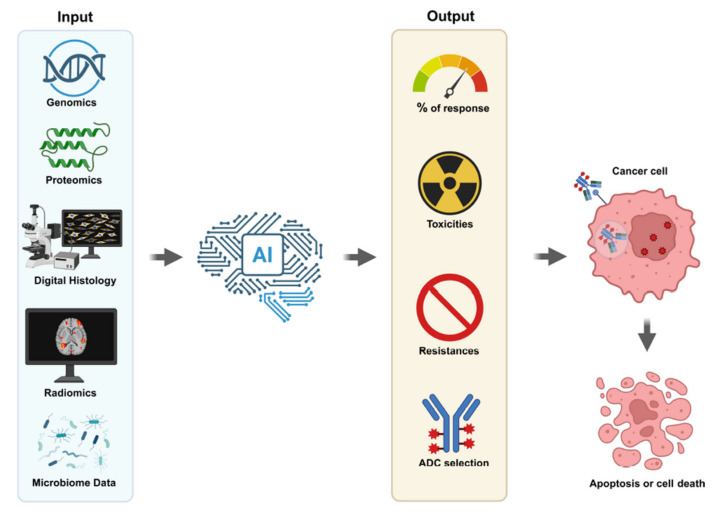
Artificial Intelligence Antibody Drug Conjugate Selection. Understanding the right ADC with lower toxicity and resistance and increased response to cancer could be screened with AI. The system’s success strictly depends on the quality of data from genomics, proteomics, digital histology, radiomics and microbiome assays.

Precision oncology has revolutionized cancer treatment, tailoring therapies to individual tumor profiles. Among the recent advances, Antibody-Drug Conjugates (ADCs) have emerged as a promising class of biopharmaceutical drugs combining the targeting capabilities of monoclonal antibodies with the potent cytotoxic effects of chemotherapeutic agents [1]. Despite their success, predicting patient response to ADCs remains a significant clinical challenge [2]. Recent developments in artificial intelligence (AI), particularly machine learning (ML) and deep learning (DL), offer new opportunities to enhance predictive capabilities, optimize patient selection, and improve outcomes (Figure 1).

The revolution in AI has significantly energized the medical research field, particularly in improving the prediction of therapy efficacy. The strong desire to enhance the accuracy of therapy predictions using AI has driven the development of new models, making them more applicable in clinical settings such as breast cancer detection [3]. However, the U.S. Food and Drug Administration sometimes had to support AI-based prognostic tools that ultimately produced inaccurate predictions for specific patients at risk of heart failure. Given the medical research community’s occasional unrealistic expectations regarding AI advancements and their potential medical applications, it is crucial to implement standard procedures for AI-based cancer models. These models must adhere to general parameters for standardization, transparency in their logistic modules, and avoidance of algorithm biases [4,5,6].

In this review, we summarize the current knowledge about AI-based prognostic methods and discuss their potential future applications in predicting the efficacy of antibody-drug conjugates in cancer patients, highlighting current methodologies, challenges, opportunities, and future directions.

## 2. Antibody-Drug Conjugates: Mechanisms and Clinical Challenges

ADCs are complex molecules composed of three key components: a monoclonal antibody, a cytotoxic payload, and a linker that connects these two components [7]. Their selective targeting ideally minimizes systemic toxicity and maximizes therapeutic efficacy. Notable examples include trastuzumab emtansine (T-DM1) or trastuzumab deruxtecan for HER2-positive breast cancer and sacituzumab govitecan for triple-negative breast cancer (TNBC) [8].

However, several factors complicate ADC efficacy prediction:

### 2.1. Tumor Antigen Heterogeneity

One major limitation stem from tumor antigen heterogeneity, which refers to the uneven presence of the target protein across cancer cells. Because ADCs rely on binding to specific antigens, any variation in expression, whether between different tumors, within the same tumor, or over time, can result in incomplete targeting [9]. Some tumor cells may not express enough antigen to be effectively reached by the ADC, allowing them to evade treatment [10]. Additionally, cancer cells can reduce antigen levels as a survival strategy in response to therapy, further complicating treatment outcomes [10].

### 2.2. Intracellular Trafficking Variability

The process by which ADCs enter and move within cancer cells, known as intracellular trafficking, also varies and impacts drug effectiveness. After binding to the target antigen on the cell surface, ADCs must be internalized and transported to the lysosome, where the linker is broken down to release the cytotoxic drug. Variations in this pathway, such as differences in the speed of internalization, the efficiency of lysosomal degradation, or diversion into recycling pathways, can lead to less drug being released inside the tumor cells, reducing therapeutic impact [11].

### 2.3. Drug Resistance Mechanisms

Another challenge comes from drug resistance mechanisms that tumors can develop or already possess. Tumor cells may pump the cytotoxic drug out using efflux transporters, alter the pathways that trigger cell death, or enhance their ability to repair the damage caused by the drug. These adaptations reduce the susceptibility of cancer cells to the ADC payload [12]. Resistance can also arise if the tumor modifies intracellular enzymes required for linker cleavage or if it becomes cross-resistant to similar chemotherapy agents, limiting the overall effectiveness of the treatment [13].

### 2.4. Tumor Microenvironment Influences

The tumor microenvironment—the complex network of non-cancerous cells, extracellular matrix, and blood vessels surrounding the tumor—can significantly impact ADC delivery and action. Factors such as dense stromal tissue, poor blood supply, and high pressure within the tumor can restrict how deeply the ADC penetrates the tumor mass [14]. Furthermore, immune cells within the microenvironment may unintentionally absorb the ADCs, decreasing the amount available to target cancer cells and potentially causing off-target effects [15]. Immune-suppressing components of the microenvironment can also weaken any immune system involvement that might support ADC activity [9].

These diverse challenges highlight the necessity for advanced approaches that integrate biological and clinical information to better predict patient responses to ADC therapy. Such strategies are essential to optimize treatment plans and fully harness the potential of ADCs in personalized cancer care. Consequently, there is a critical need for tools that can integrate multi-omic and clinical data to predict ADC response accurately.

## 3. AI in Oncology: A Paradigm Shift

AI technologies, particularly ML and DL, have demonstrated remarkable success in oncology, from image analysis to genomics interpretation. Key advantages include:

- Ability to manage large, complex datasets;
- Identification of non-linear patterns;
- Continuous model improvement with increasing data.

In the context of ADCs, AI can assist in patient stratification, biomarker discovery, and real-time prediction of therapeutic response.

As AI has been unleashing its true potential, the field of medicinal oncology has been scrutinizing a continuous but inevitable paradigm shift in the way patients are diagnosed and treated. Given the infinite variations proteins can have since their origin 3.7 billion years ago, and unlimited possible interactions with other molecules exist, new methods were needed to predict their structures. X-ray crystallography was originally used to determine protein structures, a process that takes from a few months to several years. After the COVID-19 pandemic, an AI program developed by DeepMind AlphaFold was able to predict three-dimensional protein structure from amino acid sequences with very high accuracy [16]. This breakthrough awarded the Nobel prize in chemistry in 2024 to Demis Hassabis and John Jumper. AI technologies, particularly ML and DL, have demonstrated remarkable success in oncology, from image analysis to genomics interpretation. They can manage large, complex datasets to improve the accuracy of predictions.

Medical misdiagnosis is a serious issue. According to a study at Johns Hopkins Medicine, every year, more than a third of a million patients die and more than four hundred thousand suffer from permanent disabilities in the U.S. because of an incorrect diagnosis [17]. AI could offer more accurate diagnostic methods, reducing the human cost associated with patient loss on families, and reducing the cost of inappropriate prescriptions of drugs.

AI’s potential to manage large, complex datasets to identify non-linear patterns has been proven in radiology. In contrast to traditional image-based diagnostic procedures, AI-aided methods can improve diagnostic performance. Combining radiomics and AI is capable of successfully extracting and processing large multidimensional data from cancer images, which could be easily interpreted by AI to give very detailed suggestions for a life-saving route of action. The original data is generated from computed tomography (CT) scans, ultrasound (US), magnetic resonance imaging (MRI), X-ray imaging, and digital subtraction angiography [18]. In the field of oncology, coupling AI with radiomics has been able to improve hepatocellular carcinoma characterization and prognosis when compared to traditional radiological methods. This coupling facilitated the determination of intricate relationships between variables from radiomics and clinical outcomes [19]. In radiomics, Volume of Interest (VOI) is a crucial step restricting X-ray regions to a specific anatomical target. Currently, VOI has been manually delineated. ML was implemented to automatically do it in radiomics analysis, by the natural language processing method of automatic text segmentation [20,21]. Furthermore, conventional steps of ML radiomics analysis can be bypassed with DL. Starting from unprocessed HCC cancer images, DL could produce an outcome through filtering and calculation steps. Further, the convolutional neural networks can improve their logistics through active learning, enhancing their overall accuracy and prediction capability [22]. Remarkably, time is a variable that can be incorporated in the image lesion enhancement patterns evaluation process [23,24].

Data scaling improves AI models in oncology. It is important to detect tumors such as breast cancer early, when treatments can still be very successful [25]. Google’s AI systems have improved mammogram performance by using datasets from multiple institutions for training purposes. An AI model exists that is capable of surpassing human experts in predicting breast cancer. In a Nature paper, McKinney et al. reported that in contrast to traditional systems, Google’s algorithm could detect more breast cancers with fewer false positives and false negatives from images. Evaluating mammograms of 91,000 women in both the US and the UK, Google’s system lowered the false negatives rate by 6% in the US and by 1.2% in the UK, and false positives by 9% in the US and by 3% in the UK [26].

Large-scale diverse datasets can generate more accurate multimodal AI. Current AIs operate on a single modality, neglecting the broader clinical context. A wide spectrum of modalities exists in oncology, from imaging (CT, MRI, PET), histology, genomics, to lab and health electronic narrative records. Integrating data from different modalities allows for increasing the robustness and accuracy of the diagnostic and prognostic models [27]. After ADCs reach the market, AI can analyze real-world data (RWD) and electronic health records (EHRs) to monitor effectiveness and rare toxicities in diverse patient populations. These insights can feed back into the development pipeline, informing future iterations of ADC design and post-marketing risk mitigation strategies.

Overall, DL is superior to ML in many aspects, even if it requires more training. A compelling rationale for implementing AI in ADC response prediction is implementing DL with data scaling. ADC efficiency depends on antigen expression levels (e.g., HER2, B7-H3, CD19), payload sensitivity, internalization and trafficking within the cells, and the tumor microenvironment. Combining the available large omics data regarding the ADCs with patients’ diagnostic images, histology could help build a multimodal AI.

## 4. AI Methodologies for Predicting ADC Response

The prediction of therapeutic response to ADCs has become a critical challenge in precision oncology. As ADCs grow in clinical relevance, AI and ML methods are increasingly being employed to model their complex biological interactions and predict patient-specific outcomes. A variety of AI approaches have emerged, each leveraging different data modalities and computational strategies to improve prediction accuracy. We provide here a brief review of these approaches and an outlook on future developments.

### 4.1. Classical Machine Learning Approaches

Early efforts to predict ADC response employed traditional ML algorithms, including random forests, support vector machines (SVMs), and gradient-boosted decision trees. These models typically relied on handcrafted features derived from molecular descriptors, gene expression profiles, or clinical metadata. While relatively interpretable and straightforward to implement, their performance was often constrained by the quality and dimensionality of the input features. Although these models were trained on only a few hundred ADCs, they achieved respectable performance metrics, with area under the ROC curve (AUC) values ranging from 0.70 to 0.80. However, their accuracy plateaued, as fixed descriptors fail to capture complex interactions among antigen affinity, intracellular trafficking, and drug release kinetics. Despite these limitations, such models remain useful as rapid screening tools and performance benchmarks for more advanced methods, though they rarely generalize well to novel chemotypes or previously uncharacterized target antigens.

### 4.2. Deep Learning Approaches

Deep learning [28] has significantly advanced the field by enabling automatic feature extraction from high-dimensional data. Convolutional Neural Networks (CNNs) and Recurrent Neural Networks (RNNs) have been applied to genomic sequences, histopathological images, and time-series clinical data. These models can capture complex non-linear relationships but require large datasets and careful tuning to avoid overfitting.

Further advancement is represented by message-passing neural networks (MPNNs) [29], a type of graph neural network (GNN) that learns to represent graph-structured data by iteratively passing, aggregating, and updating messages (i.e., information) between nodes in the graph. In MPNN, payload molecules are represented as graphs in which atoms are nodes and bonds are edges. DumplingGNN [29], a hybrid architecture that stacks MPNN, graph attention, and GraphSAGE layers [30], parses both 2-D topology and 3-D conformations, achieving an accuracy of more than 91% on a specialized payload set while retaining interpretability through attention maps that pinpoint toxicity-driving substructures.

### 4.3. Large Language Models

Large language models (LLMs) are the state-of-the-art applications of AI, increasingly utilized across diverse contexts and representing a primary driver of AI integration into everyday life. In essence, an LLM is a complex deep network-based algorithm trained on extensive textual data to comprehend and generate human-like language [31].

LLMs pretrained on millions of antibody or antigen sequences have changed the granularity at which one can model binding and internalization. The approach known as ADCNet [32] is an emblematic example: it combines ESM-2 embeddings for antibody/antigen sequences with FG-BERT embeddings for SMILES strings of linkers and payloads, plus the DAR as a scalar feature, in a unified transformer encoder. On a curated 8000 ADC dataset, it reached 0.93 AUC, outperforming every classical ML baseline and offering the first web server dedicated to ADC activity prediction. A key advantage of these models over traditional ML approaches is their strong ability to generalize to previously unseen sequence variants.

LLMs are also increasingly being tailored to the adaptive immune repertoire. Llama-Affinity [33], developed on the Llama-3 architecture and trained on over 2 × 10^9^ observed antibody repertoire sequences, predicts antibody–antigen binding with an AUC of approximately 0.99, while reducing training time by a factor of five.

### 4.4. Integrative Models

However, true ADC response depends not only on the drug but also on the biological context into which it is delivered. Attention-guided multi-omics integration (AGMI [34,35,36]) grafts cell-line gene-expression, mutation, and methylation data onto a multi-edge graph and uses a graph-edge-aware network to forecast IC_50_ values, yielding 8–34% gains over earlier multi-omics baselines. More recently, scGPT embeddings of single-cell expression profiles [37] have been plugged into the DeepCDR architecture [38], producing state-of-the-art performance across multiple tumor types and highlighting the value of foundation models for capturing subtle lineage-specific drug-resistance programs. Transfer-learning ideas from TransCDR [38] and other oncology pipelines show that embeddings trained on unrelated small-molecule screens can be frozen and adapted to ADC datasets with minimal labeled examples, mitigating the perpetual data scarcity issue in this context. Moreover, these examples clearly demonstrate that hybrid models integrating clinical data, imaging, and multi-omic profiles, via ensemble learning and multimodal architectures, show strong potential in capturing the complex, multidimensional nature of ADC response mechanisms.

### 4.5. Future Developments

Over the next few years, the field is likely to converge on digital-twin pipelines that iterate between in silico simulations and bench experiments in real time. This paradigm will enable the resolution of some of the most persistent challenges in ADC development. In particular, the integration of spatial transcriptomics and multiplexed immunofluorescence layers will allow AI models to reason about drug penetration gradients within solid tumors, one of the key failure modes of current ADCs. In addition, payload-generative GNNs, pre-trained on cytotoxic compound libraries, will incorporate reward signals from rapid cell viability assays streamed directly from cloud laboratories, closing the design-test-learn loop in hours instead of weeks. Finally, federated learning (FL) frameworks will allow multi-center consortia to train ADC response predictors on private patient datasets without exchanging raw data, thereby enhancing cohort diversity while complying with data-sovereignty regulations.

In summary, the field has moved considerably beyond handcrafted features, embracing foundation model embeddings and graph-based reasoning. The most powerful systems now integrate molecular, protein, and cellular modalities into unified, multimodal frameworks. Within just a few years, it is reasonable to expect that AI-guided ADC discovery will transition from retrospective prediction to real-time, interpretable design-accelerating development timelines and expanding the therapeutic window for complex and heterogeneous cancers.

## 5. Data Sources and Integration

### 5.1. Clinical Trial Databases

Data from ADC-focused clinical trials provide valuable resources for training predictive models, including survival outcomes, adverse event profiles, and biomarker status. As the number of available ADCs grows alongside evolving treatment algorithms, efforts to optimize ADC dosing, treatment duration, and rechallenge strategies have become a priority in the field [39]. Clinical trial datasets that encompass efficacy, toxicity, and biomarker data enable AI models to stratify patient subgroups that are most likely to benefit with the least adverse effects or outcomes. Large-scale analyses of ADC trials have demonstrated that combining biomarker status with survival and toxicity data enhances patient selection and stratification, which are tasks that AI can perform more efficiently and at a greater scale than traditional methods [40,41,42,43,44]. Studies show that merging molecular and clinical datasets reveals predictive patterns of treatment response, reinforcing the utility of these datasets in AI-driven modeling. Ascione et al. emphasized that determinants of ADC efficacy are not only limited to the presence of target antigens but are also shaped by the varying quantitative expression thresholds, receptor mutations, and the heterogeneous influence of the tumor microenvironment on bystander cytotoxicity [45]. These layers of complexity necessitate the use of AI, which can integrate high-dimensional data such as in vitro antigen expression levels, intratumoral heterogeneity, resistance mechanisms, and off-target protein effects [46]. AI models can be trained on these multi-parametric datasets generated from clinical trials, high-throughput screening, and real-world data to provide optimized patient stratification and uncover functional relevance of ADC targets, especially when standard binary biomarker thresholds are insufficient.

Clinical trials such as DESTINY-Breast03 and DESTINY-Breast04, which evaluated trastuzumab deruxtecan in HER2-positive and HER2-low metastatic breast cancer, respectively, report comprehensive survival, toxicity, and HER2 biomarker data to support subgroup identification for ADC therapy [47,48,49,50]. Similarly, the TROPiCS-02 trial of sacituzumab govitecan offers deep clinical and biomarker profiling, all of which can be leveraged in predictive modeling [51]. Beyond survival metrics, the DESTINY-Breast04 trial also showed that trastuzumab deruxtecan preserved global health status and quality of life over a longer treatment period compared to a chemotherapy regimen and treatment from physician choice alone, with significantly delayed deterioration across multiple patient-reported outcomes, including outcomes such as pain and physical functioning. These results underscore the multidimensional therapeutic value of ADCs and the databases that guide their use. As future trials incorporate increasingly granular clinical, molecular, and patient-recorded outcome datasets, AI models offer the ability to synthesize these layers to predict not only biological response but also patient tolerability and quality of life [52,53]. Ongoing trials such as DESTINY-Breast09 are expected to provide first-line efficacy and biomarker data for trastuzumab deruxtecan combinations, which will further expand the clinical datasets available for AI integration. Clinical trial datasets that integrate efficacy, toxicity, and biomarker data along with long-term patient outcomes and more granular subsets of data support the application of AI-driven approaches to optimize treatment strategies and patient selection in ADC therapy.

### 5.2. Multi-Omic Datasets

Genomic, transcriptomic, proteomic, and single-cell data enhance the understanding of tumor biology influencing ADC efficacy. Available resources, such as The Cancer Genome Atlas (TCGA), can provide valuable insights into mRNA, protein, and overall genetic expression, in tandem with correlated patient outcomes. The use of transcriptomic and proteomic data can be utilized to prioritize and validate ADC targets across different patients and cancers [54]. Single-cell multi-omics builds on this by resolving intratumoral heterogeneity, mapping antigen expression at the cellular level, and characterizing the tumor microenvironment which all play a key role in predicting ADC response and resistance [55,56,57]. A pan-cancer analysis by Bosi et al. integrated mutation status, tumor microenvironment heterogeneity, gene or protein expression, and antigen profiles across more than 9000 tumors, further highlighting the role of AI in refining ADC target selection and clinical optimization [54]. Potential combination strategies can be revealed with the analysis of multiple target co-expression within specific subsets of patients, possibly uncovering resistance mechanisms such as payload metabolism genes or other downstream effectors influencing outcomes [55,56,57]. The integration of these datasets has enabled the discovery of novel biomarkers, and with the potential of predictive models that are primed for AI use case in estimating ADC response, future oncology precision efforts can be maximized [58,59,60,61]. Altogether, the convergence of multi-omic and single-cell technologies provide a more complete view of tumor biology, guiding smarter ADC design, target selection, and therapeutic strategies. These tools are rapidly transforming ADC development by offering functional insights into antigenicity, heterogeneity, and resistance that directly inform clinical outcomes. Although multi-omics integration has emerged as a powerful approach to enhance ADC response prediction, it is not without limitations. Different omics layers contribute unevenly to predictive performance, and some modalities may introduce redundancy, noise, or even overfitting, potentially diminishing model reliability [62]. Evidence from ablation studies and information gain analyses suggests that transcriptomic and single-cell expression data frequently provide the most informative features, whereas methylation profiles or less-characterized microbiome layers often add limited incremental value [63]. To mitigate these challenges, strategies such as feature selection, modality-specific weighting, and noise reduction are increasingly applied, ensuring that only the most predictive signals are incorporated. By critically evaluating the contribution of each omics layer, AI models can achieve a balance between leveraging complementary biological information and maintaining robustness, interpretability, and clinical applicability, ultimately supporting more reliable and actionable predictions for ADC therapy.

### 5.3. Imaging Data

Radiomics and digital pathology images offer non-invasive predictive biomarkers, with deep learning models extracting high-dimensional features correlating with response. Radiomics involves the quantitative analysis of medical images (e.g., CT, MRI, PET) to capture phenotypic tumor traits [64]. Imaging modalities are non-invasive tools for predicting treatment response in oncology. In recent years, researchers have used AI to extract features from imaging data that correlate with clinical outcomes [65]. When processed through deep learning models, these features can help to classify histological and molecular subtypes, stratify patients by their supposed risk, and predict responses to therapies such as chemotherapy and immunotherapy [66,67,68]. Deep learning models, particularly convolutional neural networks, can automatically extract complex features from both radiology and digital pathology images [69,70]. These representations often reflect tumor heterogeneity, spatial patterns, and microenvironmental architecture which all paint a thorough clinical picture on patient prognosis and diagnosis. In digital pathology, deep learning enables direct extraction of clinically meaningful biomarkers from routine histology slides, supporting tasks like molecular feature inference and end-to-end therapy response prediction, even going as far as predicting RNA-Seq expression of tumors from whole slide images [71,72]. While challenges remain such as reproducibility, standardization, and prospective validation, the integration of radiomics and digital pathology with AI is advancing precision oncology. These tools hold strong potential for improving individualized treatment planning and patient outcomes, along with clinician involvement.

### 5.4. Microbiome Data

Emerging evidence suggests that gut and tumor microbiomes modulate immunotherapy and potentially ADC efficacy, providing an additional predictive layer. While the role of the microbiomes in immunotherapy is well established, recent studies suggest that microbial composition and function can also affect ADC pharmacodynamics, toxicity, and resistance through immune modulation, drug metabolism, and alterations in the tumor microenvironment [73,74,75]. Dysbiosis has been linked to changes in drug response and increased toxicity in both solid and hematologic cancers. Specifically, certain microbial taxa have been shown to impact immune activation and treatment outcomes [76]. Microbiome data being explored as a set of predictive biomarkers for ADC efficacy is an emerging concept, and evidence remains largely preclinical, especially in the context of ADCs. AI and machine learning tools are being applied to large-scale microbiome, genomic, and clinical datasets to identify microbial signatures and host-microbe interactions that correlate with treatment response or resistance [77,78,79]. Advanced AI models, including digital gut twins and multi-omic integration frameworks, are being developed to simulate individual responses and predict how microbiome changes may influence ADC outcomes [77,80,81]. These approaches enable more precise patient selection, supporting the design of more effective ADCs tailored to specific patient microbiota, and guide microbiome-based strategies to improve efficacy and reduce toxicity. While these models show promise for precision oncology, their implementation is limited by data and regulatory challenges, and as such, they are not yet standard in clinical practices. As this field advances, the combination of microbiome research, ADC development, and AI-driven analysis is expected to uncover new biomarkers and therapeutic strategies that improve clinical outcomes in cancer patients.

## 6. Challenges and Limitations

In the field of ADCs, AI may be implemented in multiple steps, including patient stratification, biomarker discovery, and prediction of therapeutic response [44]. However, AI implementation in clinical practice faces several challenges and limitations that are discussed below.

### 6.1. Data Heterogeneity and Scarcity

The effective training, validation and optimization of AI models relies on the continuous availability and supply of high-quality and well-annotated datasets across multiple institutions, without compromising patient privacy and intellectual property [82]. Despite FL, heterogeneity in sample handling, technological platforms, source data, imaging protocols and data labeling can introduce biases [83]. Additionally, AI models may display limited performance when analyzing data from diverse patient cohorts [84], underscoring the importance of training and validating AI models using diverse demographic, ethnical and socioeconomic groups prior to their implementation [85,86].

### 6.2. Model Interpretability 

AI models, particularly those based on deep learning, rely on extensive datasets and sophisticated model architectures comprising millions of parameters. This level of complexity poses significant challenges in discerning which parameters contribute to the decisions and endpoint predictions of a particular model [87]. Consequently, AI models are often perceived as ‘black boxes,’ potentially imposing trust and utilization barriers among clinicians, researchers, and patients [87]. Therefore, understanding the decision-making processes within AI models is essential for enhancing transparency and is likely to facilitate their integration into clinical workflows [88]. Overall, initiatives aimed at educating medical professionals and physicians on the benefits, risks, and limitations of AI models within their respective fields are essential and hold significant potential to enhance the adoption of AI-based technologies in clinical practice [87].

### 6.3. Generalizability 

In the context of AI models, generalizability is defined as the capacity of a model to maintain robust performance when applied to novel cohorts and datasets that differ from those used during training and validation stages [89]. To optimize the generalizability of AI models, it is recommended that patient cohort selection be conducted in an unbiased manner, ensuring representation of diverse patient populations and incorporating multi-center validation [85,86,89]. Additionally, the field is evolving towards the implementation of guidelines with well-defined frameworks aiming at maximizing generalizability [90]. These include Minimum Information for Medical AI Reporting (MINIMAR) [91], Standard Protocol Items: Recommendations for Interventional Trials-Artificial Intelligence (SPIRIT-AI) [92], Consolidated Standards of Reporting of Trials-Artificial Intelligence (CONSORT-AI) [93], Developmental and Exploratory Clinical Investigations of DEcision support systems driven by Artificial Intelligence (DECIDE-AI) [94], and Generative Artificial intelligence tools in Medical Research (GAMER) [95].

### 6.4. Regulatory and Ethical Considerations 

The rapid progress of AI within the healthcare space has led to the emergence of novel tools and applications that frequently outpace existing regulatory mechanisms. While these innovations hold considerable promise, they simultaneously present complex ethical and regulatory challenges, underscoring the need for oversight and policy development in this domain [96,97,98]. The utilization of patient data in AI-driven models raises significant concerns regarding privacy, informed consent, fairness, safety, transparency and accountability, thereby necessitating the development of robust ethical frameworks [96,97,98]. The World Health Organization (WHO) published a document containing regulatory considerations for the use of AI in health [99]. This important resource discusses key topics, including documentation, AI development lifecycle, risk management, analytical and clinical validation, data quality, patient privacy and collaboration [99].

## 7. Future Directions

### 7.1. Federated Learning 

FL allows model training across institutions without sharing sensitive patient data, overcoming data scarcity and privacy concerns. FL is a decentralized machine learning method to train a model across multiple centers, without the need to share any local data. The data is stored at each local device or server (also known as the client), instead of being sent to the central server, for training. Only model updates are shared with the central server. Model updates consist of batch gradient descent, stochastic gradient descent, and mini-batch gradient descent.

The advantages of FL are the focus on privacy and security, essential aspects for patients’ confidential information, by keeping sensitive data on local devices. Differential privacy could be used to further anonymize the data, introducing another layer of privacy. Federal averaging aggregates the data, which is sent to the central server to improve the global model. The clients can participate at different times, and the method could be scaled to millions of devices. This model allows learning from a large variety of clinical data across borders that otherwise may not be accessible.

Challenges of this method are data heterogeneity, high intensity of data ending up straining a central server, time for decentralization, and convergence. Heterogeneity can be resolved by a normalization step before data submission to the central server, even and filtering only those with high quality. Besides Apple and Google, healthcare institutions have been using these AI models for medical diagnostics. At least thirty-two articles have been published on the clinical applications of FL [100]. Access to crucial computing resources remains an issue, exacerbating unfairness in participation in resource-limited institutions. These institutions may receive suboptimal models, recapitulating a knowledge gap that could be otherwise filled. Having more complete data would benefit everyone, rich and limited institutions alike. A framework has been recently proposed to dynamically adjust to varying computational capacities, ensuring fair participation [101].

### 7.2. Explainable AI

Developing interpretable models (e.g., SHAP values, attention mechanisms) will enhance clinical trust and facilitate regulatory approval. With XAI, decisions and behaviors of the AI can be easily explained to humans. It has the potential to transform healthcare by making AI-driven medical decisions transparent, reliable, medically and ethically compliant [102]. Among other advantages are the increase in trust, accountability, and fairness. It can help developers to find and improve potential errors, vulnerabilities, and biases in the model, which would ultimately enable its faster refinement. Adding humans to the loop permits us to override any potential errors or biases of AI, making the XAI more socially accountable.

Notwithstanding this potential, there is a trade-off between accuracy and interpretability with the complexity of the model, such as using deep learning. It is more difficult to standardize the method, and it can lead to an oversimplification. To address these challenges, there is a need for interdisciplinary collaboration, creating common guidelines across institutions. New systems would be tailored to keep deep learning high level of complexity, while making it easy to interpret. In the field of oncology, XAI has been used to identify different cancer-causing lesions through medical imaging data [103]. Gradients have been employed to identify the molecular, cellular, and microenvironmental features that drive model predictions, including antigen expression levels, payload properties, and tumor heterogeneity. Beyond technical interpretability, these approaches can be paired with visualization tools, summary metrics, and clinician-friendly dashboards to present model outputs in a format that supports informed decision-making and patient stratification. By highlighting the most influential predictors and providing transparent rationales for model recommendations, XAI not only improves trust and accountability among clinicians but also facilitates responsible integration of AI into ADC selection and precision oncology workflows.

### 7.3. Integration of Microbiome Insights 

Incorporating microbiome profiles into AI models may reveal novel modulators of ADC response and improve predictive performance.

The gut microbiome profile has been considered the second genome. Its metabolites can modulate therapeutic outcomes in cancer patients [104]. The microbiota can affect the absorption, distribution, metabolism, and excretion ADME of drugs, altering their therapeutic efficacy and toxicity [105]. On the other hand, drugs can change the composition of microbiota, changing their metabolism and immune response. The microbiome could also metabolize payloads or linkers, the microenvironment through microbial metabolites, or inflammation. It can interact with liver enzymes, modulating the clearance of systemic drugs. Composite scores derived from the microbiome are its α/β diversity, dysbiosis indexes from qPCR assays, functional pathways, short-chain fatty acid, and bile acid metabolism. In training AI models, the correlation between microbiome patterns and ADC success/failure could be accessed. The prediction would be validated with external or longitudinal cohorts.

### 7.4. Real-Time Adaptive Models 

Continuous learning from real-world data and clinical feedback loops will allow models to adapt dynamically, enhancing precision over time. This technology has been continuously explored in the field of oncology, where it is critical to respond to new data and patient conditions over time. Such models can continuously learn from genomic data, tumor progression, and response to therapy. With their aid, it would be possible to switch treatment based on how cancer is responding. An application is the use of machine learning algorithms to detect minimal residual disease (MDR) in acute myeloid leukemia (AML) and myelodysplastic syndrome with multicolor flow cytometry. The algorithm showed promising accuracies (84.6–92.4%; *n* = 5046) in predicting better progression-free survival and overall survival for AML (*p* < 0.0001) [106]. MR-EDGE, an AI-powered method, might be able to detect circulating tumor DNA (ctDNA) in the blood of cancer patients with unsurpassed sensitivity to predict cancer recurrence. In one instance, MRD-EDGE was able to detect five colorectal-cancer recurrences without any false negatives [107]. Similarly, MRD-EDGE showed sensitivity in detecting early-stage lung cancer and triple-negative breast cancer patients and tracking tumor status during treatment. Technology could even detect mutant DNA from precancerous colorectal adenomas.

These encouraging results show that AI could improve oncology to detect cancer even from premalignant lesions, a significant breakthrough in the field of oncology.

## 8. Discussion 

ADCs have emerged as a transformative class of therapeutic agents in oncology, particularly in the treatment of solid tumors. By combining the selectivity of monoclonal antibodies with the potent cytotoxic effects of chemotherapy, ADCs offer a highly targeted therapeutic strategy. The recent success of agents like trastuzumab deruxtecan (T-DXd), sacituzumab govitecan, and enfortumab vedotin in breast, urothelial, and lung cancers has not only reinvigorated interest in this therapeutic platform, but also demonstrated the clinical potential of ADCs beyond the traditional targets and tumor types. Despite these successes, ADCs also face significant limitations. On-target off-tumor toxicity, limited payload penetration in solid tumor masses, heterogeneous antigen expression, and resistance mechanisms such as antigen downregulation and efflux pump activation represent ongoing challenges. Interstitial lung disease (ILD), a concerning adverse event with T-DXd, exemplifies the need for improved safety monitoring and predictive biomarkers.

The ADC development pipeline is expanding rapidly, with numerous agents in clinical and preclinical stages targeting novel antigens such as HER3 (patritumab deruxtecan), Nectin-4 (enfortumab vedotin), B7-H3 (MGC018), TROP-2 (datopotamab deruxtecan), and LIV-1 (ladiratuzumab vedotin). These agents are being explored not only in breast and lung cancer but also, in gastrointestinal, gynecological, and head and neck malignancies. Emerging technologies are also enabling the development of bispecific ADCs, dual-payload ADCs, and conditionally activated ADCs. These advances aim to increase tumor specificity, broaden the therapeutic window, and reduce systemic toxicities. Additionally, improvements in linker chemistry and payload potency are further enhancing the stability and effectiveness of newer generation ADCs.

In this new, exciting, and promising scenario, AI is poised to revolutionize the development, optimization, and clinical deployment of ADCs. Given the complexity of ADCs, which combine monoclonal antibodies, cytotoxic payloads, and linkers into a single therapeutic entity, AI offers a powerful set of tools to accelerate discovery and enhance precision at every stage of the pipeline. AI algorithms, particularly those leveraging ML and deep learning, are increasingly used to model and simulate the structural and functional interactions of ADC components. These tools can predict optimal antibody–antigen binding affinities, identify targetable tumor-associated antigens with minimal off-target effects, and evaluate linker stability and payload release mechanisms. For instance, generative AI models have shown promise in designing novel cytotoxic payloads with improved selectivity and lower systemic toxicity.

AI-based data mining of multi-omics datasets, including genomics, proteomics, and single-cell transcriptomics, can uncover novel cancer-specific antigens suitable for ADC targeting. By integrating data across thousands of tumor samples, AI can identify biomarkers predictive of ADC efficacy or resistance, enabling a more personalized and stratified treatment approach. AI tools can analyze preclinical and clinical datasets to model pharmacokinetics (PK) and pharmacodynamics (PD), predict on-target and off-target toxicity, and identify patient populations most likely to benefit from specific ADCs. This approach can inform go/no-go decisions in early development and support adaptive clinical trial designs. Predictive algorithms may also guide optimal dosing regimens to maximize therapeutic windows while minimizing adverse effects. AI-integrated platforms are now being developed to automate the screening of thousands of ADC combinations.

By combining robotics, imaging, and AI-driven analysis, these systems can evaluate cell viability, internalization efficiency, and cytotoxic responses at an unprecedented scale and speed. Such platforms shorten the timeline from target identification to clinical candidate selection. Emerging biotech companies and academic groups are developing closed-loop systems where AI continuously learns from experimental data, refines molecular models, and proposes new ADC candidates in a cycle of rapid iteration. This “self-improving” approach could significantly reduce development timelines and costs while expanding the therapeutic potential of ADCs to new tumor types and indications.

Recent advances in ADC design have led to the emergence of novel format conjugates, including bispecific ADCs, dual-payload constructs, conditionally activated ADCs (e.g., Probody ADCs), and nanobody-based ADCs [108]. These next-generation formats aim to enhance tumor specificity, overcome heterogeneous antigen expression, and improve therapeutic windows. However, they introduce additional layers of complexity that may challenge conventional predictive models, which often assume linear relationships between a single-antigen expression and drug response. To accommodate these architectures, AI models may require fundamental adaptations, such as multi-target modeling, integration of inter-payload interaction dynamics, or graph-based representations that capture complex molecular and cellular interactions. Incorporating these strategies into predictive pipelines could enable accurate patient stratification and response prediction for next-generation ADCs, highlighting the need for continuous evolution of AI approaches alongside innovations in ADC design [109,110].

Despite the promise that AI holds for advancing the development of ADCs, several important challenges and limitations must be addressed to fully realize its potential. Here are some perceptions: (1) Regarding the Data Quality and Availability, AI and machine learning models are only as good as the data they are trained on. In the context of ADCs, high-quality, well-annotated datasets that link molecular features to clinical outcomes are often limited, fragmented, or proprietary. Sparse datasets in rare tumor types or novel ADC payloads hinder generalizability and can lead to biased models. Additionally, inconsistencies in experimental methodologies and reporting standards across studies complicate data integration; (2) The effectiveness of ADCs depends on a delicate balance of factors—target expression, internalization rates, linker cleavage, payload potency, immune response, and tumor microenvironment. Modeling this multidimensional biology with sufficient accuracy remains a major hurdle, especially when relying solely on in silico simulations or incomplete biological inputs; (3) Deep learning models often lack transparency, acting as “black boxes”. XAI is an emerging solution, but still at its early stage; (4) The integration of AI into drug development introduces complex regulatory questions. It remains unclear how regulators like the FDA or EMA will assess and validate AI-generated designs, predictions, or recommendations. Questions of accountability, bias, and reproducibility are particularly relevant in high-stakes areas, such as oncology. Moreover, patient data privacy and compliance with data protection laws (e.g., GDPR, HIPAA) are ongoing concerns in the use of AI for biomarker discovery and clinical data mining; (5) biopharmaceutical companies and academic groups often develop their own proprietary AI models using custom pipelines. The lack of standardized, validated AI tools for ADC design limits cross-institutional collaboration and slows down translational progress. There is a pressing need for harmonized platforms and open datasets to support collective advancement; (6) successful adoption of AI requires integration into existing drug discovery and development workflows, which are often rigid, siloed, and highly regulated. Bridging the gap between computational predictions and wet-lab validation requires close collaboration between AI experts, medicinal chemists, pharmacologists, and clinical researchers, an interdisciplinary effort that is still evolving in many institutions; (7) developing and training AI models, especially for deep learning on large, complex biological data, requires substantial computational resources and specialized talent. For small biotech firms or academic centers, these requirements may be prohibitive without external partnerships or dedicated funding streams. (8) Although several AI-based models have demonstrated a promising predictive performance for ADC efficacy, most remain at the preclinical or retrospective validation stage. The majority of studies rely on limited or single-institution datasets that do not fully reflect the heterogeneity of real-world patient populations in terms of tumor biology, treatment history, and demographic diversity. As a result, these models often exhibit reduced generalizability when applied outside their original development context. To enable meaningful clinical translation, future studies should incorporate external and multi-institutional validation cohorts as well as prospective clinical datasets. The integration of real-world data—including electronic health records, imaging repositories, and genomic or proteomic profiles—will be essential to improve model robustness and ensure reproducibility across diverse clinical settings. Moreover, establishing collaborative frameworks between clinicians, data scientists, and pharmaceutical researchers could accelerate the design of AI-guided clinical trials, where model predictions are evaluated in real time to guide ADC selection or dosing strategies. Demonstrating consistent performance, clinical utility, and patient benefit through such studies will be the critical next step toward deploying AI-driven tools as part of routine clinical decision-making in ADC therapy. (9) A key challenge for current AI models in predicting ADC response is their limited generalizability across diverse tumor types, antigens, and ADC architectures. While many models report high predictive accuracy within their training datasets, their performance often diminishes when applied to previously unseen molecular entities or patient cohorts, reflecting the intrinsic heterogeneity of tumor microenvironments and the structural diversity of ADCs. Addressing this limitation requires strategies such as transfer learning, domain adaptation, and multi-task learning, which enable models to leverage knowledge from well-characterized datasets and adapt to novel domains with minimal labeled data. Additionally, the integration of large, multi-center, and multi-omic datasets, encompassing genomic, proteomic, imaging, and clinical features, can further enhance robustness and reduce overfitting. Establishing standardized benchmarking protocols and performing external validation on independent cohorts are also essential steps to ensure that AI predictions remain reliable and clinically actionable across different ADC types, tumor contexts, and patient populations. These measures collectively support the development of AI tools that are not only accurate but also generalizable, interpretable, and suitable for real-world clinical implementation.

To harness the full potential of AI in ADC development, the field must adopt a dual approach: technological innovation paired with strategic alignment to clinical, regulatory, and ethical standards. Future efforts should focus on:

Creating shared, standardized, high-quality datasets for ADCs and associated clinical outcomes. Building transparent and explainable AI systems that satisfy regulatory scrutiny. Establishing multi-disciplinary consortia to bridge data science, oncology, and regulatory science. Promoting open science and data sharing to accelerate collective progress.

## 9. Conclusions

ADCs are poised to become a cornerstone of precision oncology. As newer agents with better specificity, reduced toxicity, and expanded indications enter clinical practice, they hold the potential to redefine therapeutic strategies for multiple solid tumors. The incorporation of AI into ADC development and clinical deployment represent a transformative step forward, enabling a new era of personalized, data-driven cancer care. Future clinical trials investigating this concept are warranted. With careful management of these challenges, AI can become an integral partner in the next generation of ADC discovery and development, enabling smarter, faster, and more patient-centric oncology therapeutics. Moreover, the strategic investment, interdisciplinary collaboration, and patient-centered research would support the AI integration with ADCs, fundamentally altering the trajectory of cancer treatment in the coming decade.
